# Patient Segmentation: Adjust the Production Logic to the Medical Knowledge Applied and the Patient's Ability to Self-Manage—A Discussion Paper

**DOI:** 10.3389/fpubh.2020.00195

**Published:** 2020-05-27

**Authors:** Mats Brommels

**Affiliations:** Department of Learning, Informatics, Management and Ethics, Karolinska Institutet (KI), Solna, Sweden

**Keywords:** patient segmentation, health care, production logic, patient self-management, service co-production

## Abstract

This discussion paper argues that population segmentation according to healthcare needs and risks—the usual approach—might help to identify patients for targeted action, but does not inform how to design efficient service delivery. In other service industries customer segmentation is typically done based on customer preferences. Products or services are customized and marketing strategies designed to reach the most profitable customers and improve revenue generation. This paper presents an alternative approach, in which patient needs are matched with a production logic derived from the medical knowledge needed to manage the health problem, and patients' willingness and ability to self-manage and co-produce services. Seven segments are identified: healthy persons; persons with incidental needs; persons with chronic conditions; persons with multiple health problems and illnesses (often elderly); persons needing precise elective interventions; persons needing qualified accident and emergency services; and tertiary care patients. Designing care to suit these patient segments will use resources more efficiently, with better prospects of favorable medical outcomes, a higher service quality, less complications, and improved patient safety.

## Patient Segmentation in Healthcare Is Based on Patient Characteristics Only

The health services research literature deals mostly with patient segmentation from a population perspective. The ambition is to divide the population into mutually exclusive, homogeneous groups based on similar needs for services, and organizing care adjusted to meet those needs (1). In some instances, the aim of patient segmentation is to design targeted care models and intervention programmes, tailored to the needs of specific patient groups (2). Assessing risk for ill-health dividing the population into risk-strata is central to many segmentation efforts. High-risk patients who also are frequent users of healthcare services and thus costly to the healthcare system are of specific interest (3). This small group is typically said to be small, <10% of the population, but yet utilizing up to 80% of healthcare resources. Targeted efforts to reduce the need for services among these people would reduce human suffering as well as the burden to the healthcare system.

Patient segmentation is often discussed in relation to integrated care (1, 2). In those systems, attention is paid to ensuring that patients with multiple needs receive coordinated services from all relevant providers in healthcare, but increasingly also social and community care. With the increasing complexity arising from the need to coordinate an increasing number of actors, targeted efforts to ensure that patients receive “seamless care” will benefit from the segmentation of patients into groups with reasonably well-predicted service needs.

Vuik et al. (2) propose a framework for patient segmentation in integrated care by separating whole population approaches (macro-level) from subpopulation analyses (meso-level) and the identification of high-risk patients (micro-level). Subpopulations typically are people with long-term conditions or frail elderly patients. High-risk patients are identified on the individual level in order to enable targeted care planning and case management. Risk is typically expressed in terms of risk of high service utilization and thus healthcare expenditure, and insurance claims data are used to calculate and predict risk (3).

Patient segmentation is usually based on the following elements: the assessment, definition, and operationalisation of *population or patient characteristics* that are related to healthcare needs, *outcomes* aimed at when addressing population or patient needs, and the *segmentation logic* expressing how subpopulations, or patient groups are formed.

Individual needs are related to risk of morbidity or mortality, usually defined by a condition or diagnosis, pain, discomfort, functional status, social support needed, dysfunction, and frailty, but also social and behavioral health needs (1, 3, 4). Those medically defined characteristics are associated with demographic factors like age and gender as well as socioeconomic conditions and lifestyles (1, 3).

Outcomes included in reported patient segmentation schemes typically focus on service utilization: reduction of emergency service visit, hospital admissions, and readmissions as well as costs. Those are also looked upon as proxies of clinically meaningful outcomes, more closely related to the medical condition. More direct outcome measures are reduced morbidity or alleviated symptoms (1–3).

The segmentation logic may be “completely data-driven” or “non-completely data-driven.” In the first case, data from a number of sources are pooled and statistical clustering is performed on those datasets. In a non-completely data-driven approach, expert input is used for defining segmentation criteria (1). Those data are qualitative, based on clinical judgement, as are some data on health status, symptoms, and lifestyles acquired from (electronic) health records (3).

Dana et al. (3) explored how US healthcare delivery organizations with risk-based funding segment their “high need, high cost” patients. They found that predictive analytics based on quantitative data (like claims data) was insufficient, and that segmentation required additional information from clinical assessments. Risk scores would not differentiate between patients with varying clinical, social, or behavioral health needs.

Chong et al. (1) performed a systematic review of population segmentation systems based on need. They found 16 studies that categorized patient populations and demonstrated an operationalised segmentation scheme. Eleven of these were “expert-driven” and five “data-driven.” In four cases the segmentation was predominantly based on medical assessments, in three cases on utilization, and in nine cases on a mix of medical, demographic, and utilization indicators. Eleven of the segmentation systems had 4–8 segments, three 10–20 segments, one 92 segments, and one 6–269 segments. The two with the biggest number of categories are used to predict clinical severity or service utilization on an individual level, and are less useful as segmentation tools.

The London Health Commission segmentation of the population of London, reported by Vuik et al. (2) is partly data-driven. A database covering 200,000 Londoners presented data on health, social, and community care utilization, costs, diagnoses, and patient level characteristics. In a decision-tree analysis characteristics like health problems and age were found to be predictors of total cost, and thus suitable for segmentation. Eight conditions (mostly healthy, one or more long-term condition, cancer, severe mental illness, learning disability, advanced dementia, socially excluded groups) and four age groups (0–12, 13–17, 18–64, 65, and older) were cross-tabulated, producing 15 distinct segments.

Researchers at the Center for Medicare and Medicaid Services, US Department of Health and Human Services, criticized a frequent approach, also used by other service industries, to “stratify a customer population into groups that are sufficiently homogenous to enable arranging a set of commonly needed supports and services to meet their expected needs” (5). They claim that such an approach leads to segments only linked to the service providers that customers presently use. Examples would be “nursing home clients” or “home help clients.” As an alternative they propose a segmentation that is based on patients' “health prospects and priorities.” The latter undoubtedly introduces an element of “patient-centredness,” which is a goal service providers increasingly strive to meet. The authors do not describe the process used to derive a total of eight patient population segments, but those obviously represent a consensus view of a number of experts consulted over a period time, as “comments from scores of our colleagues over several years have helped share the ideas” (5). Three “considerations” guided their process, and those are worth citing as useful principles to apply when dividing patient populations into segments with the intention to use those in planning services:

1. The number of segments need to be limited
2. The segments need to include everyone meeting the segmentation criteria
3. The people in each segment should have similar healthcare needs, rhythms of needs, and priorities—and segments need to be sufficiently discriminatory.

The patient segments proposed by Lynn et al. (5) are: healthy; maternal and infant health; acutely ill; chronic conditions, normal functions; stable but serious disability; short period of decline before dying; limited reserve and exacerbations; frailty, with or without dementia. The authors go on to define requirements on the health care and support process for each segment that would ensure that the services offered meet the six quality criteria of the Institute of Medicine (6), that is, safe, timely, effective, efficient, equitable, and patient-centered.

In summary, patient population segmentation systems presented in the literature mostly focus on stratifying patients by assessed needs or risks, they are “data-driven” with adjustments done by clinical experts, and aim at targeting patients, like “heavy service users,” for preventive action or tailored care plans. In some instances the systems are intended to support the provision of seamless care to patients with complex needs by integrating services and coordinating an array of inputs of service providers.

## Customer Segmentation in Service Industries

In other service industries, typically operating in competitive markets, customer segmentation is done based on the analysis of customer *preferences*. The information is used to customize products or services as well as designing marketing strategies (7). The overall objective is to improve revenue generation (8) and increase customer loyalty (9). In one study with that approach customers were divided into “rationalists,” “functionalists” or “value maximisers” (10).

Insights from marketing and customer relations management, briefly reported above, have also been used in healthcare, in order to “understand attitudes and behaviors to attract, retain, and engage consumers” (11). Segmentation of patients is then based on answers to attitudinal and behavioral questions, not demographic, service utilization or clinical data. A survey among US patients identified four different types of consumerist behavior in healthcare:

- Bystanders, who are complacent, reluctant to use technology and unwilling to change
- Trailblazers, tech-savvy, self-directed, and engaged in wellness activities
- Prospectors, who rely on recommendations from friends and family and see providers as trusted friends
- Homesteaders, a group that is reserved and cautious and can be looked upon as traditionalists.

This segmentation is useful especially for service providers wanting to identify persons who are willing to take responsibility for their own health and use new technologies for self-care and telehealth, freeing human resources that providers can use to approach less confident persons and offer traditional support.

Finally, operations management, more specifically supply-chain management, uses customer segmentation in “advanced planning systems” (APS) (12). An APS aims at finding “feasible, near optimal plans across a supply chain as a whole, while potential bottlenecks are considered explicitly” [(13), cited in (12)]. It matches constraints as to material and capacity with customer orders. Customers are divided into hierarchical segments (based on buying behavior, strategic value, and profitability) and the limited resources are allocated to customers in the order of their importance, thus optimizing overall business performance.

Process thinking, originally introduced as a part of quality management in health care, raised the interest in applying operations management approaches to service planning and provision. Patient pathways and “flows” across provider and unit borders were described and analyzed. “Lean approaches” have been widely implemented in healthcare with the intention to streamline and standardize patient processes and remove waits and waste in order to increase efficiency, improve quality, and to reduce complications (14).

Emergency departments create separate flows or *service lines* for patient segments to deal only with their specific needs, thus minimizing waiting times and avoiding unnecessary resources being kept idle. Those service lines are, i.a., “fast tracks” for patients with minor illness in need of advice only, acute care/resuscitation and trauma units with multi-professional staff for severely ill, and “main ED” for patients needing investigations and observation (15).

## A New Approach: To Match Patient Characteristics With the Service Production and Distribution Logics

Based on the reviews presented above most work on patient segmentation explore and operationalise patient needs and risks and aim at designing care plans or “service packages” to meet those needs. No attention is given attempts to find a fit between patient needs and the production logic of the services that best meet the needs. Neither does one find a discussion on what service distribution channels are most suitable for that production logic. In addition, a patient's interest, willingness, and ability to self-manage the health problem will have an impact on the production logic. I will in the following present a proposition based on those three elements and discuss its rationale and design.

Medical knowledge is applied to characterize and categorize health problems (into diagnoses) and choose treatments to deal with the problems. The nature of that knowledge base defines the resulting patient care process. Bohmer (16) emphasizes that health problems cover a continuum from highly unstructured and uncertain to highly structured with high certainty, and that the diagnostic and therapeutic processes have to be adjusted accordingly. Unstructured problems will require probing, that is a trial and error approach, whereas highly structured ones are managed by rules application. Ideally there would be a progress in the knowledge base over time, moving problem-solving toward more problems being more specific enabling an algorithmic treatment approach. Bohmer also makes the case that when the medical knowledge base is firm—the diagnosis is accurate and the treatment is well-established—then the care process is *sequential*, and *evidence-based medicine* can be practiced. That sequential process resembles an assembly line. When the diagnosis is unclear, or when there is no clear-cut guideline to follow, the approach is (consecutive) probing, leading to an *iterative process*. Bohmer (16) calls this activity a *job shop*.

In the same fashion Christensen et al. (17) relate the care provider business model to the “maturity” of the relevant medical knowledge base. Referring to Stabell and Fjeldstad (18) they distinguish between “precision medicine” which will enable an algorithmic approach, which they call a *value-adding process*. When the knowledge base is uncertain, a situation they refer to as “intuitive medicine,” the business model is a *solution shop*. When patients have chronic conditions and multi-morbidity they are dependent on a number of different service providers producing a coordinated service—which according to Christensen's terminology requires a *facilitated network* of providers. The two first ones are identical to Bohmer's assembly line and job shop.

By applying the reasoning of Bohmer (16) and Christensen et al. (17) three different production logics, dependent on the nature of the health problem, and the medical knowledge used, will apply to health service provision: solution shop, value adding process, and facilitated network. Services are either distributed face-to-face in one suitably equipped facility or at a distance enabled by information and communication technology, and possibly, robotics.

## A Patient Segmentation Proposal

As pointed out by Lynn et al. (5) the number of patient segments needs to be limited in order for the segmentation system to be practical. The following proposal consists of seven patient segments. The *segmentation logic* is based on (i) the production logic of the professional service related to how well the health problem is structured and supported by specific medical knowledge; (ii) the service distribution channel (service distributed in physical proximity or at a distance utilizing e-health tools); and (iii) the capability and interest of patients to self-manage their health and disease. The segmentation logic and the benefits achieved by tailoring services accordingly is presented in Table 1.

**Table 1:** Seven patient segments: segmentation logic.

**Patient segment**	**Production logic**	**Distribution channel**	**Degree of patient self-management**	**Value added**
Healthy persons	On-demand health promotion and prevention advice and services	E-health tools and health kiosks	Very high	Personal well-being
Persons with incidental needs	Solution shop with nurse practitioners and generalist back-up	E-health consultations and GP office	High	Easy access, timely service
Persons with chronic conditions	Diagnosis-specific solution shop with nurse practitioners and specialist support	E-health tools Patient self-monitoring and shared decision-support Diagnosis-specific outpatient offices	High	Continuity of care Improved clinical outcomes and quality of life Service co-production and off-loading healthcare providers
Persons with multiple illnesses (Frail elderly)	Facilitated network of providers Case manager	Personalized services by multiple mobile providers E-health surveillance Residential homes	Medium to low	Integrated care and seamless services
Persons needing precise elective interventions	Value-added process (organized as standardized patient pathways)	Diagnosis/procedure specific short-stay units (focused factories)	Low	Resource-efficiency Timeliness and high safety
Persons needing accident and emergency services	Solution shops with tracks according to patient urgency and risk	Specialized acute care facility with hospital back-up	Low	Timeliness Good clinical outcomes High patient safety
Tertiary care patients	Solution shop with super-specialists	Hospital with high-tech equipment	High, medium or low	Solutions to highly complex problems

### The Patient Segments

#### Healthy Persons

These persons are provided information about healthy lifestyles and encouraged to engage in those. They are offered preventive services like vaccinations and screening programmes for the early detection of potentially severe diseases. These services require a single intervention and are distributed over the internet and health kiosks in easily accessible locations like pharmacies and shopping centers.

#### Persons With Incidental Needs

Persons with occasional non-threatening health problems like common colds are offered distance-consulting over the internet and, when needed, visits to a drop-in facility at a primary care provider. Those providers are working as solution shops, operated by nurses with physician back-up, applying pre-designed decision algorithms.

#### Persons With Chronic Conditions

These persons self-manage their disease with the support of self-tracking and measurement tools, linked to a disease registry. They communicate with the healthcare provider electronically when the disease is stable. When exacerbations occur, problems are dealt with over the net, or by visiting the provider. Shared decision-making on treatments utilize the registry by applying predictive modeling. The result is service co-production with a solution shop approach when problems arise.

#### Persons With Multiple Health Problems and Illnesses (Often Elderly)

These patients need a network of health and social care service providers, coordinated by a case manager.

#### Persons Needing Precise Elective Interventions

The intervention needed is provided as a value-added process, applying a predefined patient pathway. Services are mostly offered in short-stay facilities. Service providers specialize on one or a few similar processes, performed in a “focused factory.”

#### Persons Needing Qualified Accident and Emergency Services

Accident and emergency services are organized in hospital facilities that offer all diagnostic and acute care services needed. Patients are further segmented according to urgency and risk. Highly competent staff meets patients at admission to identify those requiring immediate life-saving interventions, those needing urgent treatment, those to be directly admitted to in-hospital care, and easily solved cases (“see-and-treat” or fast-track patients).

#### Tertiary Care Patients

These are patients with complex health problems, rare diseases, or conditions where treatment has to be centralized because of need for specific expertise or expensive technology. The service is organized as a solution shop.

## To Conclude

The rationale of this segmentation approach is to avoid that patients are managed only by one production logic, which usually is the solution shop, leading to the development of excess capacity and diseconomies of scope. By differentiating the health problems, and choosing a service production process accustomed to patients' needs and actions to meet those as derived from medical knowledge, and, in addition, utilizing patients' abilities to self-manage, and co-produce services, resources are more efficiently used, resulting in better prospects of favorable medical outcomes, a higher service quality, less complications, and improved patient safety.

## Data Availability Statement

All datasets generated for this study are included in the article/supplementary material.

## Author Contributions

The author confirms being the sole contributor of this work and has approved it for publication.

## Conflict of Interest

The author declares that the research was conducted in the absence of any commercial or financial relationships that could be construed as a potential conflict of interest.
